# Symptoms and clinical parameters of pediatric and adolescent migraine, by gender - a retrospective cohort study

**DOI:** 10.1186/s10194-017-0789-z

**Published:** 2017-08-08

**Authors:** Tal Eidlitz-Markus, Avraham Zeharia

**Affiliations:** 1 Pediatric Headache Clinic, Day Hospitalization Department, Schneider Children’s Medical Center of Israel, Petach Tikva, Israel; 2 Sackler Faculty of Medicine, Tel Aviv University, Tel Aviv, Israel

**Keywords:** Female, Males, Migraine, Pediatric, Adolescent, Chronic migraine, Age onset, Migraine with aura, Puberty

## Abstract

**Background:**

The available data on gender differences in clinical migraine parameters among pediatric patients are based on relatively few studies, which did not use the current version of the International Classification of Headache Disorders (ICHD) of the International Headache Society. The aim of the present study was to compare between males and females, demographic and clinical characteristics of children and adolescents with migraines diagnosed according to the ICDIII-beta version.

**Methods:**

The electronic database of a tertiary pediatric headache clinic was searched for all children and adolescents diagnosed with migraine headaches in 2010–2016. Data on demographics, symptoms, and headache-related parameters were collected from the medical files. Findings were compared by gender.

**Results:**

The cohort included 468 children and adolescents of mean age 11.3 ± 3.6 years; 215 males (45.9%) and 253 females (54.1%). Migraine without aura was documented in 313 patients (66.9%), and migraine with aura in 127 (27.1%); 28 patients (6.0%) had probable migraines. The female patients had significantly higher values than the male patients for the following parameters: age at admission (*p* = 0.042, Cohen’s *d* 0.8303, 95% CI 0.614–0.992); age at migraine onset (*p* = 0.021, Cohen’s *d* 0.211, 95% CI 0.029–0.394); rate of migraine with aura (OR 2.01, 95% CI 1.29–3.16, *p* = 0.0056); headache frequency (*p* = 0.0149, Cohen’s *d* 0.211, 95% CI 0.029–0.3940); rate of chronic migraine (*p* = 0.036, OR 1.54, 95% CI 1.02–2.34); and puberty (OR 3.51, 95% CI 2.01–6.35, *p* = <0.001). Males had a higher rate of vomiting (OR 0.62, 95% CI 0.41–0.93, *p* = 0.018). Further analysis by pubertal stage revealed that pubertal females, but not prepubertal females, had a significantly higher rate of migraine with aura than did males (41.1% versus 28.9%; OR 1.42, 95% CI 0.85–2.37, *p* = 0.039).

**Conclusion:**

Female children and adolescents with migraine treated in a tertiary pediatric headache clinic were characterized by a higher rate of chronic migraine and migraine with aura, a lower rate of vomiting, and older age at onset relative to males. These findings might be influenced by the better description of migraine symptoms by females owing to their better verbal ability.

## Background

Worldwide data show that migraines are two to three times more prevalent in women than in men [1]. In addition, clinical symptoms of adult migraine differ by gender, with women having a longer attack duration, greater intensity of headache attacks, higher rate of headache recurrence, greater disability, and a longer recovery time [2, 3]. Nausea, phonophobia and photophobia, and aura were all reported to be more frequent in women [3]. Differences were also demonstrated in brain high-field magnetic resonance imaging between adult female and male migraineurs, and between female migraineurs and healthy controls [4].

In children, migraines were found to occur at a similar rate in both genders before the age of 10 years but was more common in girls after the age of 11 years [5]. Accordingly, high-field magnetic resonance imaging studies of children and adolescents with migraine showed both gender and developmental differences in brain anatomy and structure compared to healthy controls [6]. However, the clinical data on the pediatric age group are derived from relatively few studies, all based on older versions of the International Classification of Headache Disorders (ICHD1) of the International Headache Society (IHS) [7, 8].

The aim of the present study was to compare between males and females, demographic and clinical parameters in a large sample of male and female pediatric migraineurs diagnosed according to the 2013 ICHD III-beta version [9].

## Methods

### Patients and setting

The electronic database of the headache clinic of a tertiary, university-affiliated, pediatric medical center was searched for all children and adolescents diagnosed at ages 3–18 years with migraine headache during 2010–2016. The diagnosis was re-evaluated in all cases according to ICHD-III beta version [9]. Exclusion criteria were headache type other than migraine.

The study was approved by the Research Ethics Committee of Rabin Medical Center (approval no.RMC-pp10–16). Owing to the retrospective design of the study, the committee waived the need for informed consent.

### Headache clinic protocol

At the first visit to the headache clinic, children and their parents, or only the parents of very young children, are interviewed by the headache clinic physician using a questionnaire based on the ICHD-IIII beta version. Questionnaire items include the frequency, duration, and nature of the headache attacks, time from the start of the headaches to presentation at the clinic, and related symptoms. If a discrepancy arises between the reports of a child and his /her parent(s), the anamnesis is extended by requesting both the parent and the child to repeat the description of the headache episodes and other symptoms in greater detail. This also ensures that the child understands the questions. For very young children with limited verbal communication, headache frequency and symptoms are determined by the child’s complaints and the parents’ impression from the child’s behavior (according to the criteria of the ICHD-III beta version) For patients who were admitted before 2013, their medical files were reviewed and migraine was diagnosed according to the ICHD-III criteria [9]. In addition, parents are questioned about their diagnosisgiven by of the parents’ community physician, their own medical history of migraine, and migraines in other family members (yes/no; diagnosed/not diagnosed by a physician). The final diagnosis in each case is only reached after extensive follow-up, with a review of all the findings of all the patient’s visits at the headache clinic.

### Study procedure

Data on demographics, headache symptoms, and other clinical headache-related parameters were collected from the medical files of the patients who were found eligible to participate in the study. The findings were compared between male and female patients.

### Statistical analysis

With the proposed sample size of 210 for each of the two groups (males/females), the study would have 80.3% power to yield a statistically significant result. This computation assumes that the difference in proportions is −0.12 (specifically, 0.20 versus 0.32).

Data were analyzed using BMPD software (University of California Press, Los Angeles, CA, 1993). Continuous variables were calculated as means and standard deviations and compared between groups using analysis of variance ANOVA. Parameters that did not have a Gaussian distribution were compared between groups using the nonparametric Mann-Whitney U-test. Discrete variables were calculated as numbers and percentages, and compared between groups using Pearson’s chi-square or Fisher’s exact test, as applicable. A *p* value of ≤0.05 was considered significant. Stepwise logistic regression was used to identify factors with a significant effect on gender differences in migraine-related parameters. The odds ratio (OR) and 95% confidence intervals (CI) were determined. The effect size for continuous variables was calculated with Cohen’s *d.*

## Results

The database search identified 468 children and adolescents with migraines, 215 boys (45.9%) and 253 girls (54.1%), who attended the pediatric headache clinic during the study period. Mean age at admission to the headache clinic was 11.3 ± 3.6 years (range 3.8–18, median 11.1), and at onset of headache disease, 9.0 ± 3.8 years (range 3.25–17.92, median 8.7). The mean interval from onset of headache disease to presentation at the clinic was 26.6 ± 26.4 months (range 1–124, median 18). Migraine without aura was documented in 313 patients (66.9%), and migraine with aura, in 127 (27.1%); the remaining 28 patients (6.0%) had probable migraines. Mean headache frequency was 13.5 ± 11.4 headaches per month (range 0.03–30, median 8); 155 patients (33.11%) had chronic migraines (≥15 episodes/month) and 298 (63.6%) had episodic migraines. Fifteen patients did not report headache frequency. Psychiatric comorbidities were documented in 94 patients (20.08%), organic comorbidity was reported in 53 patients.

Compared to children older than 6 years, children younger than 6 years had lower mean headache frequency 9.31 ± 11.31 vs 14.03 ± 11.32 per month, *P* < 0.001; lesser mean duration of headaches before admission 11.26 ± 8.79 vs 28.63 ± 27.27 months, *P* < 0.001; and similar mean duration of headache attacks 12.88 ± 17.2 vs 12.52 ± 15.11 h, *P* = 0.77.

Table 1 compares clinical parameters between male and female patients. The female patients had significantly higher values of the following parameters: age at admission (*p* = 0.042; Cohen’s *d* 0.8303, 95% CI 0.614–0.992), age at migraine onset (*p* = 0.021; Cohen’s *d* 0.211, 95% CI 0.029–0.394), rate of migraine with aura (*p* = 0.0056; OR 2.01, 95% CI 1.29–3.16), headache frequency (*p* = 0.0149; Cohen’s *d* 0.211, 95% CI 0.029–0.3940); rate of chronic migraine (*p* = 0.036; Cohen’s *d* 1.54, 95% CI 1.02 2.34), and rate of puberty (*p* < 0.001; OR 3.51, 95% CI 2.01–6.35). The males had a significantly higher rate of vomiting (*p* = 0.018; OR 0.62, 95% CI 0.41–0.93). Further comparison of the rate of migraine with aura by pubertal stage yielded a significantly higher rate in pubertal females than in pubertal males (41.1% versus 28.9%, *p* = 0.039; OR 1.42, 95% CI 0.85–2.37). No such difference was found in comparing prepubertal females and prepubertal males (27% vs 17.6%, *p* = 0.128; OR 0.87, 95% CI 0.5–1.52).

**Table 1:** Demographic and clinical parameters of pediatric patients with migraine (468 patients)

Parameter	Males (*N* = 215)	Females (*N* = 253)	*p value*
Age (yrs) at admission	10.8 ± 3.5	11.5 ± 3.7	0.042
Migraine age onset(yrs)	8.6 ± 3.6	9.4 ± 4.0	0.021
Migraine without aura	157 (73%)	156 (61.7%)	0.0056
Migraine with aura	43 (20%)	84 (33.2)	
probable migraine	15 (7%)	13 (5.1%)	
Headache frequency (mos)	12.2 ± 11.2	14.6 ± 11.5	0.0149
Chronic migraine	61(29.8%)	94 (39.5%)	0.036
Migraine duration			
Months	27.4 ± 27.8	26.0 ± 25.0	0.813
Hours	12.6 ± 16.1	11.3 ± 13.8	0.675
Phonophobia	172 (80.8%)	189 (76.2%)	0.258
Photophobia	156 (74.3%)	169 (71.1%)	0.221
Alodynia	44 (31.9%)	58 (36.9%)	0.391
Awakening pain	101 (49.3%)	120 (48.4%)	0.391
Nausea	129 (60.8%)	147 (59.5%)	0.775
Vomiting	87(41.45)	74 (30.5%)	0.018
Dizziness	45 (29.2%)	64 (38.3%)	0.098
Puberty	45 (20.9%)	112 (44.2%)	<0.001
Parental migraine history			
Negative paternal migraine	105(48.8%)	133(52.6%)	0.188
Paternal only	26 (12.0)	18 (7.1%)	
Maternal only	70 (32.6%)	91 (35.9%)	
Both parents	14 (6.6%)	11 (4.4%)	
Psychiatric comorbidity	44 (20.5%)	50 (19.8%)	0.908
Organic comorbidity	25(11.6%)	28(7.1%)	0.1
Preventive medical treatment	130(60.7%)	147(58.1%)	0.672

Values are presented as means ± SD or n(%)

Psychiatric comorbidity was associated with higher headache frequency,16.2 ± 12.1 headache attacks per months vs patients without psychiatric comorbidity12.8 ± 11.1, *P* = 0.01; and a higher rate of migraine with aura, 30.9% for patients with psychiatric comorbidity vs 26.2% of patients without psychiatric comorbidity (*P* = 0.02)

Table 2 shows the results of the logistic regression model. The factors that showed a significant effect on gender differences in clinical migraine-related parameters were age at admission, puberty, and diagnosis (migraine with aura).

**Table 2:** Logistic regression showing factors associated with gender (females vs males)

Variables	Odds ratio	95% CI		*p* value
		Lower limit	Upper limit	
Puberty	8.38	4.06	17.3	<0.001
Age at admission	0.834	0.762	0.914	0.001
Diagnosis (migraine with aura)	1.70	1.07	2.7	0.0221
Headache frequency per month	1.49	0.973	2.27	0.0652

Area under the curve, 0

### Missing data and excluded patients

Of the patients attending the clinic during the study period, 176 were excluded from the study because of a diagnosis of tension headache (*n* = 150) or undefined new-onset headache (*n* = 10), trigeminal cephalalgia (*n* = 3), pseudotumor cerebri (*n* = 7), headache due to head trauma (*n* = 4), and an undefined headache (*n* = 2).

Data on several parameters were missing for 58 patients. Differences in gender ratio were calculated between the patients who were missing the data and those who were not, and analyzed statistically, as follows: phonophobia, missing in 7 patients (*p* = 0.585); photophobia, 109 *(p* = 1.00); awakening pain, 15 (*p* = 0.169); paternal migraine, 8 (*p* = 1.0); maternal migraine, 7 (*p* = 0.594); nausea, 8 (*p* = 0.94), headache frequency, 25 (*p* = 1.0); vomiting, 15 (*p* = 0.44); allodynia, 173 (*p* = 1.0)’ dizziness, 147 (*p* = 0.228). None of the differences calculated was statistically significant. Therefore, after consultation with the biostatistician, we assumed the missing data had no impact on our conclusions.

## Discussion

The present study of gender differences in pediatric migraineurs shows that female patients were older than male patients at both clinic admission and onset of headache disease. Accordingly, more female patients were postpubertal at both time points; and had a higher rate of migraine with aura, greater migraine frequency, more chronic migraine, and a lower rate of vomiting.

In a large study of 2982 adults aged 18–65 years with migraine, female patients were found to be prone to a significantly longer duration of headaches and greater intensity of headache attacks than male patients, with a higher prevalence, frequency, and intensity of nausea; and a higher prevalence of phonophobia and photophobia [2].

Wöber-Bingöl et al. [7] reported on the prevalence of migraine headache symptoms in children by gender and found that the female patients had a higher frequency of migraine with aura, as in our study, and a higher rate of vomiting than the male patients, contrary to our study. Additionally, the male patients had a higher rate of phonophobia, contrary to our study. However, the study of Wöber-Bingöl et al. [7] was based on the original IHS criteria (ICHD1), whereas ours was based on the revised criteria for children of the ICHDIII-beta version. Furthermore, the age range in the earlier study was 3–19 years and in ours, 3.8–18 years.

The differences observed in the current study in migraine parameters and in symptom frequency between males and females may have an anatomical basis. This assumption is supported by brain magnetic resonance imaging studies of adult and pediatric migraineurs [4, 6]. In both age groups, gender differences were noted in neural mechanisms in regions involved in sensory, motor, and affective functions, between male and female migraineurs [4, 6]. The authors also suggested that the observed changes might be attributable to differences in the response to intermittent stress (migraine attacks) or to differential effects of gonadal hormones (testosterone versus estradiol or progesterone) on hippocampal function and brain circuitry [4, 6].

However, clinical gender-related differences in pain perception are less clear. A 2012 meta-analysis of 126 adult studies of pain perception conducted in 1998 to 2008 concluded that 10 years of laboratory research had not produced a clear and consistent pattern of gender differences in human pain sensitivity, even with the use of deep, tonic, long-lasting stimuli to mimic the clinic setting [10, 11]. Additionally, no gender differences in pain perception results were found in pediatric studies of the perception of laboratory-provoked dental pain in children (mean age 6.7 ± 2.4 years) [12] and of disease-related pain perception during acute attacks of sickle cell anemia in adolescents (median age 14.8 years) [13]. By contrast, in an evaluation of pain perception in 118 children and adolescents from grades 5 to 9, using the cold pressor task and a related pain questionnaire, Vierhaus et al. [14] found that the female subjects had significantly higher pain intensity scores by both measures. The differences among the studies may be explained by the different ages of the populations and the different methodologies used, including differences in the study settings and in the questionnaires. Such factors may also account for the findings of more pronounced temporal summation, allodynia, and secondary hypoanalgesia in women in laboratory studies [11], and for the lack of a significant difference in the rate of allodynia by gender in our pediatric survey study. Although questionnaires have demonstrated reliability in this setting, differences in the diagnosis of allodynia may have contributed to the findings [15]. Overall, the evidence supporting a possibly less efficient endogenous pain inhibitory system in females is mixed and does not necessarily apply to all pain modalities [10].

In our study, no difference of psychiatric comorbidity was found between genders. Psychiatric comorbidity was associated with higher headache frequency and with a higher rate of migraine with aura. Gender differences in response to pain were evaluated with anxiety and pain questionnaires by Fuss et al. [16] in a survey study of 1006 children aged 11.6 ± 2.7 years; of whom 27% reported having an experience of pain that lasted 3 months or longer. Girls with a history of persistent pain expressed higher levels of anxiety sensitivity (*p* < 0.001) and pain catastrophizing (*p* < 0.001) than did both girls without a history of pain and boys, regardless of pain history. The authors concluded that boys and girls appear to differ in terms of the relationship between age and pain history, and in the expression of pain-related psychological variables [16].

### Age of onset of migraine

Estrogen per se is not a cause of migraine, as males have migraines too. In addition, the evidence of a decreasing prevalence of migraine-type headache after menopause supports the involvement of factors other than estrogen [17]. Nevertheless, our finding that male patients presented earlier with migraine symptoms than did female patients is in line with the notion that estrogens, which modulate mediators and receptor systems in both the central nervous system and at the peripheral (neuro) vascular level, may act as a trigger for migraine presentation in females [4]. For example, female sex steroids enhance neuronal excitability by elevating Ca2+ and decreasing Mg2+ concentrations; and these play a role in the synthesis and release of nitric oxide and of neuropeptides; and in the function of receptors inducing vasoconstriction. These mechanisms are involved in eliciting migraines and in stimulating pain centers, respectively. The serotonergic, adrenergic and γ-aminobutyric acid (GABA)-ergic systems are also modulated by sex steroids to varying degrees, [18]. We speculate that the difference between girls and boys in the age of onset of migraines may be related to the beginning of estrogen release and migraine modulation in girls.

### Migraine diagnosis

We found that migraine with aura was significantly associated with female gender, and migraine without aura, with male gender. Similar results were reported by Wöber-Bingöl et al. [7]. The higher rate of aura in the females may be a consequence of their better neurocognitive abilities and higher verbal abilities, as observed in psychological and neuropsychological studies in adults [19]. These factors, together with the older age of our female patients, would have made it easier for them to describe migraine aura.

Aura may also be influenced by sex hormones. Studies of male and female mouse models of familial hemiplegic migraine type 1 suggest that estrogens increase susceptibility to cortical spreading depression, the pathophysiological mechanism underlying migraine aura [20]. These findings correspond to reports of an increased prevalence of migraine with aura in high estrogen states, such as during the second and third trimester of pregnancy and hormone replacement therapy [21, 22]. They are also in line with the significantly higher number of pubertal girls than boys in our cohort. In contrast, so-called estrogen withdrawal, which occurs perimenstrually, is associated with a higher prevalence of migraine without aura [21]. Accordingly, in our cohort, the rate of migraine with aura was significantly higher in pubertal females than in pubertal males. This finding was not true for prepubertal males/females.

### Chronic migraine and headache frequency

In our study, adolescent females had a significantly higher headache frequency and higher rate of chronic migraine than did adolescent males. Similarly, studies of adults reported a significantly higher recurrence rate of migraines among females than males [3], and an almost threefold higher prevalence of chronic migraine [23].

### Vomiting

Vomiting in our pediatric cohort was observed at significantly higher rates in males than females, contrary to studies in adults [24]. Since the males in our study were younger than the females, we assume their relatively worse autonomic dysfunction contributed to this difference [25].

### Parental migraine

Goodman and McGrath [26] showed that maternal modeling of pain behaviors affected their children’s own pain. Although we did not ask about other pain conditions, we found no difference in the rate of paternal migraine between male and female patients. This could explain the between-group differences in clinical parameters.

### Study limitations

This study was limited by the retrospective design and the reliance on self- and parental reports of clinical parameters of migraine for young patients. Furthermore, the sample was restricted to patients treated in a tertiary medical center, who may have had more severe disease than individuals in the general population. This might explain the high frequency of migraine attacks and high rate of chronic migraine. Moreover, the uncovered associations might simply reflect referral patterns or other idiosyncrasies of our clinic, such as the physician’s decision to refer a patient or a patient’s agreement to attend the clinic. The study may also be biased due to differences in interpretations and expectations from parents regarding their children’s behavior according to gender. Finally, a high proportion of parents failed to respond to the question on allodynia. Although there was no difference in the percentage of males and females for whom we were missing data on this matter, the overall lower number of patients may have lessened the power of the statistical analysis**.**

## Conclusions

Compared to male pediatric patients, female pediatric patients with migraines have a significantly higher rate of chronic migraine, a higher frequency of migraine with aura, older age of onset, a higher rate of onset after puberty, and a lower rate of vomiting. Some of these findings may be related to better verbal ability of females, which makes it easier for them to describe their symptoms and their pain. Estrogen levels may explain the higher rate of aura in pubertal (but not prepubertal) girls compared to boys. Further studies are needed in ambulatory settings to assess the generalizability of the findings.

## Abbreviations

ICHD: International classification of headache disorders
IHS: International headache society
